# The Role of Hypoxia Inducible Factor-1 Alpha in Bypassing Oncogene-Induced Senescence

**DOI:** 10.1371/journal.pone.0101064

**Published:** 2014-07-01

**Authors:** Mehtap Kilic Eren, Vedrana Tabor

**Affiliations:** 1 Department of Medical Biology, Adnan Menderes University Medical School and ADU-BILTEM, Aydin, Turkey; 2 Department of Medical Biochemistry and Biophysics, Karolinska Institute, Stockholm, Sweden; Boston University Medical School, United States of America

## Abstract

Oncogene induced senescence (OIS) is a sustained anti-proliferative response acutely induced in primary cells *via* activation of mitogenic oncogenes such as Ras/BRAF. This mechanism acts as an initial barrier preventing normal cells transformation into malignant cell. Besides oncogenic activation and DNA damage response (DDR), senescence is modulated by a plethora of other factors, and one of the most important one is oxygen tension of the tissue. The aim of this study was to determine the impact of hypoxia on Ras^V12^-induced senescence in human diploid fibroblasts (HDFs). We showed here that hypoxia prevents execution of oncogene induced senescence (OIS), through a strong down-regulation of senescence hallmarks, such as SA- β-galactosidase, H3K9me3, HP1γ, p53, p21^CIP1^ and p16^INK4a^ in association with induction of hypoxia inducible factor-1α (HIF-1α). In addition, hypoxia also decreased marks of H-Ras^V12^-induced DDR in both cell lines through down-regulation of ATM/ATR, Chk1 and Chk2 phosphorylation as well as decreased γ-H2AX positivity. Utilizing shRNA system targeting HIF-1α we show that HIF-1α is directly involved in down regulation of p53 and its target p21^CIP1^ but not p16^INK4a^. In line with this finding we found that knock down of HIF-1α leads to a strong induction of apoptotic response, but not restoration of senescence in Ras expressing HDFs in hypoxia. This indicates that HIF-1α is an important player in early steps of tumorigenesis, leading to suppression of senescence through its negative regulation of p53 and p21^CIP1^. In our work we describe a mechanism through which hypoxia and specifically HIF-1α preclude cells from maintaining senescence-driven anti proliferative response. These findings indicate the possible mechanism through which hypoxic environment helps premalignant cells to evade impingement of cellular failsafe pathways.

## Introduction

Healthy, normal mammalian cells are characterised by a finite replicative potential, limiting their lifespan to a finite number of divisions, finding first described by Hayflick and Moorhead in 1961 [1]. Presently known as “Hayflicks limit” it is a state of ceased cellular proliferation, where cells still retain their metabolic activity, showing changes into a more flattened morphology when compared to normally proliferating cells. This phenomenon was named cellular senescence, and can be caused by different factors such as telomere attrition, DNA damage, oncogenes, oxidative stress [2].

Experiments with ectopically expressed H-Ras^V12^ showed that when it is introduced in primary, as well as immortalized cells it induces irreversible cell cycle arrest, which was named oncogene-induced senescence (OIS) [3]–[5]. It has been shown that in human patient samples of senescent melanocyte tumors high levels H-Ras are coinciding with the senescence [6], and the same phenomenon was observed in mice, in the K-Ras^V12^-driven premalignant lung tumors [7]. OIS is a failsafe program used by cell at risk for oncogenic transformation, thereby playing a tumor suppressive role, depending on fully functioning tumor suppressors p53 and pRb, and the downstream effectors such as p21^CIP1^ and p16^INK4a^ [2].

DNA damage response (DDR) has been implicated in premature senescence induction *via* regulation of telomere attrition, but it has also been shown to mediate OIS in certain *in vitro* settings as well as in some human premalignant lesions [8]–[12]. In addition, DDR can be elicited through commonly used cytostatic drugs, and this form of senescence is called drug-induced senescence that is used as a treatment for cancer patients [13], [14].

In addition to oncogenic activation and DNA damage response, senescence is modulated by a plethora of other factors, and one of the most important ones is oxygen level present in the tissues [15]–[17]. It is important to note that most of the cell culturing conditions do not represent the true oxygen state found in the diverse tissues of the live and properly functioning organism, as most of the cell culturing is done in 20% O_2_. In contrast, in living tissues, O_2_ level are significantly lower and can range from 3–6% in the brain to 15% in the lung [18]. On the other hand, most of our knowledge of senescence is defined by the studies that have been done in hyperoxic conditions, which might contribute to induction of senescence, at least in part by induction of telomere shortening [19]. Interestingly, several studies have shown that replicative, drug- as well as oncogene-induced senescence can be prevented under lower O_2_ levels [15], [17], [19]–[21]. These studies underscore the importance of hypoxia inducible factor-1alpha (HIF-1α) in regulation of replicative and drug-induced senescence under hypoxic conditions, which is normally found in large portions of tumor tissue found in all the mammals.

HIF1 is a transcription factor, consisting of two subunits, an α subunit, which levels are oxygen dependent and β subunit that is constitutively expressed. Hydroxylation dependant binding of HIF-1α to VHL (von Hippel Lindau tumor suppressor) and its subsequent ubiquitination is possible only in the presence of oxygen. Only upon oxygen depletion HIF-1α is stabilized and heterodimerizes with HIF-1β. This heterodimer binds to HRE (hypoxia responsive elements) in promoters of many hypoxia responsive genes, which are including growth factors, angiogenic factors, anti-apoptotic factors and the factors involved in anaerobic metabolism [22], [23].

The aim of this study was to determine the impact of hypoxia on Ras-induced senescence in HDFs. For this purpose we have utilized human primary diploid fibroblasts genetically manipulated to overexpress H-Ras^V12^ oncogene and exposed them to decreased oxygen levels. Cells displayed a strong decrease in senescence markers, such as SA-β-galactosidase, H3K9me3, HP1γ, p53, p21^CIP1^ and p16^INK4a^, which are associated with induction of HIF-1α. Hypoxia also decreased marks of Ras-induced DNA damage response (DDR) in both cell lines through down-regulation of ATM/ATR, Chk1, and Chk2 as well as decreased γ-H2AX positivity. In line with this finding we showed that genetic knock down of HIF-1α restored down regulation of p53 and p21^CIP1^. Interestingly, knock down of HIF-1α leads to a strong induction of apoptotic response in hypoxic conditions whereas not restoration of senescence in the same setting, implicating HIF-1α as an important player in early steps of tumorigenesis, leading to suppression of senescence through its negative regulation of p53 and p21^CIP1^. Our findings place HIF-1α as an important modulator of oncogene, and possibly DDR induced senescence.

## Materials and Methods

### Cell Culture

Human primary fibroblasts IMR-90 (ATCC, CCL-186) and BJ (ATCC, CRL-2522) were obtained from American Type Culture Collection (ATCC) and used within 20-30 population doublings. All cells were cultured in Dulbecco's modified Eagle's medium (Gibco) plus 10% fetal bovine serum (FBS; Biochrom) and 100 Units/mL penicillin, 100 µg/mL streptomycin, 2 mmol/L glutamine. The amphotropic virus packaging cell line, Phoenix-AMPHO (ATCC, CRL-3213) was obtained from American Type Culture Collection [24]. Cells were grown in a humidified incubator under normoxic (20% O_2_) conditions at 37°C with 5% CO_2_ unless otherwise specified. Hypoxic culture conditions (1% O_2_) were achieved by using an automated humidified internal O_2_/CO_2_ incubator of a hypoxia glove box (Coy Laboratory Products, Inc.) equipped with oxygen and carbon dioxide sensors, and connected to N_2_ and CO_2_ gas cylinders. The O_2_ (1%) and CO_2_ (5%) readings were confirmed by use of an automated anaerobic monitor (Coy Laboratory Products, Inc.). After an initial exposure to low oxygen, all subsequent treatments were given within the glove box to prevent cellular damage due to re-oxygenation.

### Retroviral-Mediated Gene Transfer

H-Ras^V12^ was provided in pBABE-puro retroviral vector by Prof. Dr. Manuel Serrano. Retroviruses were packaged in Phoenix-ampho cells and concentrated as previously described [5]. Virus containing supernatants were collected at 36–48 h, supplemented with 4 mg/ml polybrene, and filtered through a 0.45-mm syringe filter. Twenty-four hours after infection, cells were expanded 1∶2 into complete media containing 1.5–2 mg/ml of puromycin. Selection in puromycin was complete within 3 to 5 days. In all experiments, the day on which a parallel plate of uninfected target cells was completely killed in selective media is referred to as day 0. IMR-90 and BJ cells were used between 20 and 30 population doubling levels. For hypoxic incubation, cells were placed in hypoxia glow box incubator immediately after puromycin selection. All hypoxic experiments were done with cells incubated 10 days post selection in hypoxic condition.

### Senescence-Associated β-Galactosidase activity

SA-β-gal activity was detected 10 days after the selection of retroviral infected cells as previously described [25], with minor modifications. At the indicated times, cells were washed with PBS, fixed with 0.5% glutaraldehyde (PBS [pH 6.0]), and washed in PBS (pH 6.0) supplemented with 1 mM MgCl_2_. Cells were incubated in X-gal working mix solution (1 mg/ml X-gal [Boehringer], 0.12 mM K_3_Fe[CN]_6_, 0.12 mM K_4_Fe[CN]_6_, 1 mM MgCl_2_ in PBS at pH 6.0) overnight at 37°C.

### Immunofluorescence analysis

Cells were grown for on cover slips either in normoxic or hypoxic incubation. Immunostaining was performed on 4% PFA-PBS fixed cells. Cells were permeabilized with 1% TritonX-PBS for 10 minutes and washed with PBS, subsequent incubation with primary antibody was performed overnight, at 4°C. The following antibodies were used: Ki-67 (clone TEC-3, M-7249, Dako), H3K9me3 (07-523, Millipore) and γH2AX (05-636, Millipore).

Cover slips containing cells were washed and incubated with secondary antibody (AlexaFluor488 goat anti-rat, AlexaFluor488 goat anti-rabbit and AlexaFluor488 rabbit anti-mouse, (Invitrogen)) in 3% BSA in PBS/0.05% Tween for one hour at 37°C in the dark. Slides were washed, and for nuclei counterstaining briefly incubated in PBS containing DAPI, mounted and analyzed.

### BrdU incorporation

BrdU incorporation assay was performed using Cell Proliferation ELISA, BrdU (colorimetric) Kit (Roche Applied Science, Indianapolis, IN) according to the manufacturer's instruction. In brief: after selection 10 days post exposure to hypoxia H-Ras^V12^ expressing cells (2000 cells in100 µl/well) were cultured in 96-well plates in complete growth media. After 48 hours, the cells were labelled using 10 µM BrdU and re-incubated overnight at 37°C in a humidified atmosphere. Cell culture media was removed 24 hours later and cells were treated with FixDenat. Next, the cells were incubated with the anti-BrdU-POD antibody for 90 minutes at room temperature. After that, the cells were washed and the substrate solution was added. The reaction product was quantified by measuring the absorbance using a scanning multi-well spectrophotometer (ELISA reader) at 370 nm with a reference wavelength of 492 nm. For comparison, we show for indicated time points the percentage of BrdU-positive Ras-expressing cells in normoxia or hypoxia relative to control (vector) cells.

### TUNEL Staining

*In Situ* Cell Death Detection kit with Fluorocein (Roche Applied Science, Indianapolis, IN) was used to label apoptotic cells. Briefly, cells were fixed in 1% paraformaldehyde (PFA) containing Triton X-100 on ice for 45 min. Cells were pelleted, rinsed, resuspended in ice cold 70% EtOH and stored at −20°C at least overnight to permeabilize prior to suspension in the TdT label/TdT enzyme mix for 1 hr at 37°C in the dark. Labeled cells were rinsed with PBS, re-fixed in 4% PFA on ice for 20 min, resuspended in PBS. According to DAPI (Invitrogen, Carlsbad, CA) staining samples were visualized by fluorescence microscopy using 20× magnification (Olympus). The percentage of TUNEL positive cells was determined using the standard formula for the apoptotic index (AI), which was calculated as follows: AI  =  (number of TUNEL-positive cells/total number of cells) x 100.

### Western Blot Analysis

Cells were washed with PBS and lysed in NP-40 lysis buffer (150 mM NaCl, 1.0% NP-40, 50 mM Tris–HCl [pH 8.0], 1 mM phenyl- methylsulfonyl fluoride, 1 mg/ml leupeptin, 1 mM sodium orthovanadate, 1 mM EDTA). Lysates were cleared by centrifugation, 10,000 rpm for 10 minutes. Samples corresponding to 50–80 µg of protein were separated on 6%, 8%, 10% or 15% SDS–PAGE gels and transferred to Immobilon-P membranes (Millipore). Western blot analysis was accomplished according to standard procedures using ECL reagent (GE Healthcare Piscataway, NJ).

The following primary antibodies were used: HIF-1α (1∶250, BD Biosciences Pharmingen, San Diego, CA), p53, p21^CIP1^, p16^INK4a^, Mif (1∶250, Santa Cruz Biotechnology, San Diego, CA, USA) and β-actin (1∶5000, T4026, Sigma). DNA damage response was analyzed using DNA Damage Antibody Sampler Kit (1∶1000, Cell Signaling Technology, Beverly, MA) including phospho-ATR (Ser428) phospho-ATM (Ser1981)(D6H9), phospho-Chk1 (Ser296) phospho-Histone H2A.X (Ser139) (20E3) phospho-Chk2 (Thr68) and. Phosphorylation of Rb protein was analyzed by Rb Ab (Ser807/811) (1∶1000, Cell Signaling Technology, Beverly, MA). HRP-conjugated secondary antibodies were purchased from GE Healthcare (Piscataway, NJ).

### Quantitative Real Time PCR

Total cellular RNA was extracted using the Qiashredder and Qiagen Rneasy Mini kits (Qiagen Inc., Valencia, CA, USA). 0,5 µg of total RNA was used to reverse transcribed into single-stranded cDNA with cDNA Synthesis Kit SuperScript III RT (Invitrogen Life Technologies, Carlsbad); gene-primers for HIF-1α and Mif were purchased from Applied Biosystems (TaqMan gene expression assay). Quantitative real-time PCR (qRT-PCR) was performed with Step One Plus Real Time PCR (Applied Biosystems, Foster City, CA, USA) instrument. The reactions were performed in triplicate and the results were normalized using Human β-actin Pre-developed TaqMan assay reagents (Applied Biosystems). Changes in the target mRNA content were determined using a comparative CT method (ABI User Bulletin number 2). The fold change was calculated using 2^−*ΔΔCt*^ (where *ΔΔCT* = *ΔCT* of *treatment*–*ΔCT* of *control*).

### RNA interference

For shRNA mediated inhibition of gene expression of HIF-1α and Mif, the following commercially available lentiviral constructs of human HIF-1α, human Mif and a negative control shRNA plasmids (sc-44225-sh, sc-37137-sh, and respectively Santa Cruz Biotechnology, San Diego, CA, USA) were used. HIF-1α, Mif shRNA, plasmid (h2) is a pool of 3 target-specific lentiviral vector plasmids each encoding 19–25 nt (plus hairpin) shRNAs designed to knock down gene expression. Each plasmid contains a puromycin resistance gene for the selection of cells stably expressing shRNA.

A concentration of 1–2 µg of each shRNA plasmid (pLKO.1 puro) was transfected into 293 T cells using shRNA Plasmid Transfection Reagent (sc-108061). Infectious lentiviruses were collected at 24 h and 48 h after transfection and the pooled supernatants were filtered through a 0.45 µm filtration unit and used to infect BJ and IMR-90 cells. After that, without any selection bulk cells were expanded and exposed to hypoxic conditions for 3 days to test for HIF-1α down-regulation. The efficiency of HIF-1α knockdown was determined by western blot and real time quantitative PCR analysis.

### Microscopy analysis

For senescence associated β-galactosidase (SA-β-gal) detection microscopy analysis was performed with the inverted bright field microscope (Olympus). Fluorescence signals were detected by fluorescence microscopy (Olympus).

For statistical analysis *student*'*s t test* was performed.

## Results

### Hypoxia prevents H-Ras^V12^- induced senescence in human diploid fibroblasts

**Figure 1:**
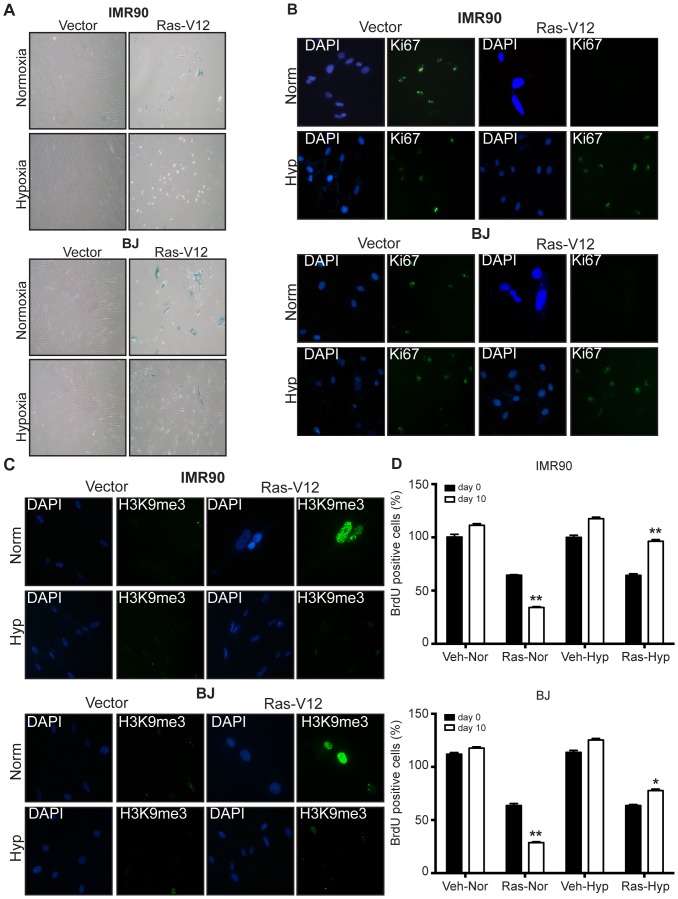
Hypoxic environment inhibits H-Ras^V12^-induced senescence in human diploid fibroblasts. Ten days post exposure to normoxia (20% O_2_, upper panel) or hypoxia (1% O_2_, lower panel) IMR-90 and BJ fibroblasts expressing control (pBabe-empty) or Ras (pBabe-H-Ras^V12^) were assessed for **A**. senescence by SA-β-gal staining, **B**. proliferation by Ki67 staining. Nuclei were counterstained with DAPI **C**. H3K9me3 staining and nuclei were counterstained with DAPI **D**. BrdU incorporation into cellular DNA during cell proliferation. V = vector, R = Ras, N = normoxia, H = hypoxia. Relative BrdU incorporation was calculated by normalization of data to values corresponding to vector (pBabe-empty) expressing cells. Statistically significant differences between Ras expressing cells in normoxia in day 0 *vs*.10 as well as in hypoxia day 0 *vs*.10 are indicated *, p<0.01 and **, p<0.05, respectively. Shown are means ± SD of 3 independent experiments in triplets.

In order to test the impact of hypoxia on Ras-induced senescence we have grown IMR-90 and BJ primary human diploid fibroblast (HDF) cells ectopically expressing H-Ras^V12^ under low oxygen conditions (1% O_2_). Ten days after culturing under hypoxic conditions we have analysed cells for senescence-associated β-galactosidase (SA-β-gal) enzymatic activity, which is a widely used standard marker of senescence. Indeed, compared to normoxia (20% O_2_) in hypoxia we observed reversal of H-Ras^V12^-driven senescence induction as shown by negative staining of the cells for SA-β-gal activity (Figure 1A). Next, in order to test the ability of non-senescent cells to proliferate in low oxygen conditions we used two different approaches: firstly, we have utilized a broad proliferation marker Ki67 (Figure 1B), and secondly we have analysed cells for their, ability to incorporate BrdU (Figure 1D and Figure S2). We found that HDFs ectopically expressing H-Ras^V12^ were positive for Ki67 antigen (Figure 1B) and incorporated BrdU (Figures 1D and S2) to a higher extent under low oxygen conditions when compared to normoxia. Overexpression of H-Ras^V12^ is known to cause accumulation of senescence-associated heterochromatic foci (SAHF) [26], areas of condensed and transcriptionally silenced DNA, which can be detected by DAPI and H3K9me3 co-staining. We also tested whether H-Ras^V12^ overexpression results in generation of SAHFs, and here we showed that SAHF formation takes place only under normoxic conditions but not when the cells were cultured under hypoxic conditions (Figure 1C). Taken together, our results suggest that H-Ras^V12^-induced senescence is blocked under low oxygen conditions, and this inhibition of senescence resulted in restoration of cell proliferative capacity of HDFs (Figures 1B and D) as evidenced by Ki67 positivity and increased incorporation of BrdU, as well as decreased senescence markers SA-β-gal, H3K9me3 and SAHFs (Figures 1A and C).

### Hypoxia down regulates senescence hallmarks and induces HIF-1α activation in HDFs

**Figure 2:**
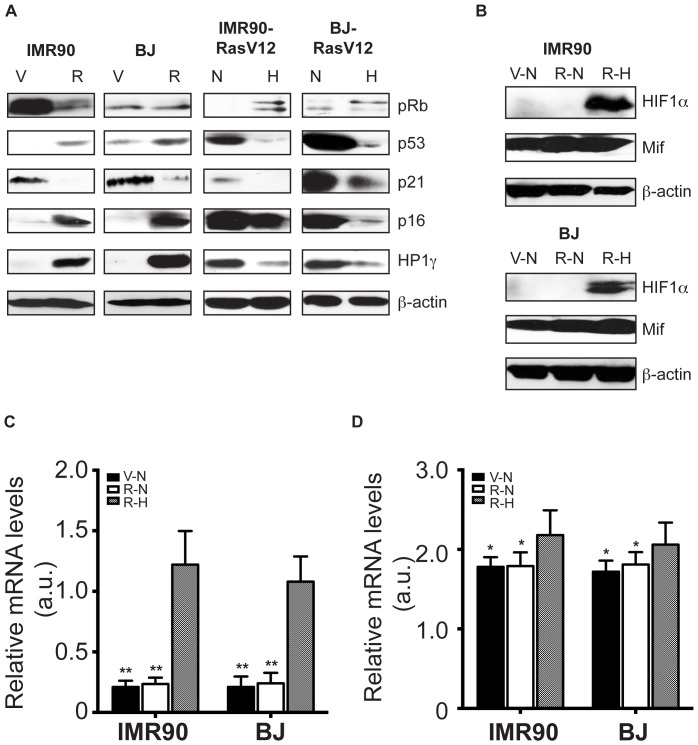
Hypoxia down regulates hallmarks of H-Ras^V12^-induced senescence and induces stabilization of HIF-1α protein. Ten days post exposure to normoxia (N, 20%O_2_) or hypoxia (H, 1%O_2_) IMR-90 and BJ fibroblasts expressing control (pBabe-empty; vehicle (V)) or Ras (pBabe-H-Ras^V12^ (R) were analyzed by **A**. Western Blotting for expression of senescence hallmarks p16^INK4a^, p21^CIP1^, HP1γ as well as the upper regulator p53 and Rb; **B**. for HIF-1α and MIF expression. β-actin was used as loading control; **C**. analyzed for the mRNA levels of HIF-1α and MIF by RT-PCR. Black bars, vehicle in normoxia, grey bars Ras in normoxia and dashed lines Ras in hypoxia. Statistically significant differences between mRNA levels of HIF-1α and Mif in Ras expressing cells in normoxia and hypoxia are indicated *, p<0.01 and **, p<0.05, respectively. Shown are means ± SD of 3 independent experiments in triplets.

In order to determine the effect of hypoxia on other hallmarks of senescence, protein extracts from H-Ras^V12^ expressing HDFs that were cultured either in normoxia or hypoxia and were analysed for the expression of p16^INK4a^, p21^CIP1^, HP1γ as well as the master regulators p53 and Rb (Figure 2A). We found that the cells grown under hypoxic conditions have reduced protein levels of all of the senescence hallmarks tested including p53, p16^INK4a^, p21^CIP1^ and HP1γ (Figure 2A). In addition, culturing under hypoxic conditions induced accumulation of phosphorylated Rb protein in H-Ras^V12^ expressing HDFs, yet another hallmark of senescence. These results indicated involvement of hypoxia in a global regulation of proteins necessary for the induction of senescence.

Next, we reasoned that if hypoxic conditions play a role in blocking of senescence it is likely that this process is dependent on hypoxia-inducible factor-1alpha (HIF-1α). Since stabilization of HIF-1α levels are not primarily dependent on H-Ras^V12^ expression (Figure 2B), but on hypoxic conditions, we tested stabilization of HIF-1α and its target macrophage migration inhibitory factor (MIF) in HDFs ectopically expressing H-Ras^V12^ both in normoxia or hypoxia. As shown by protein analyses as well as mRNA expression levels, indeed, stabilization of HIF-1α was detected in both cell lines in hypoxia but not in normoxia (Figure 2B and Figure S1). Senescence delaying effect of hypoxia in rodent cells is in part mediated through a HIF-1α and macrophage migration inhibitory factor (Mif) dependant mechanism. Thus we also assessed MIF expression in the same setting and detected a modest increase in MIF protein as well as mRNA levels under the hypoxic conditions (Figures 2B, C, D and Figure S1).

### Hypoxia-induced down regulation of p53 and p21^CIP1^ is HIF-1α dependent

**Figure 3:**
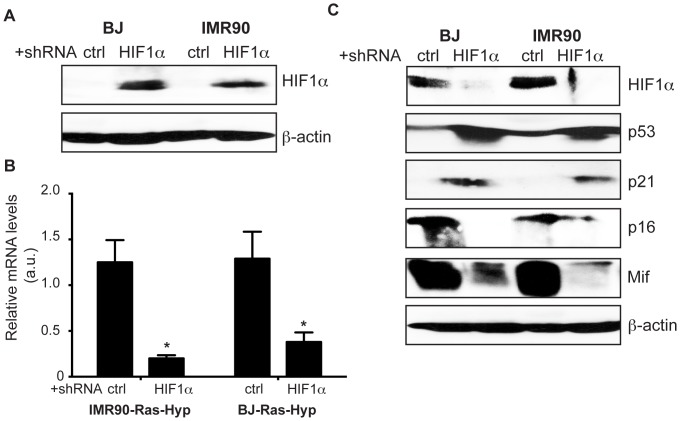
Hypoxia-driven inhibition of expression of hallmarks of senescence is Hif-1α dependent. shRNA_HIF-1α and shRNA_scr (a scrambled shRNA sequence encoding plasmid used as negative control) expressing IMR-90 and BJ cells were infected with H-Ras^V12^ and selected for puromycin for 3 days. Three days post exposure to hypoxia cells were analyzed for **A**. the expression of HIF-1α by western-blotting, β-actin was used as loading control; **B**. for mRNA level by Quantitative RT-PCR; **C**. the expression of senescence regulators p53, p21^CIP1^, p16^INK4a^ and MIF by western-blotting. β-actin was used as loading control. Statistically significant differences between mRNA levels of HIF-1α in Ras + shNC *vs*. Ras+ shHIF-1α expressing cells in hypoxia are indicated *, p<0.01. Shown are means ± SD of 3 independent experiments in triplets.

In order to investigate whether hypoxia-induced down regulation of p53, p21^CIP1^ and p16^INK4a^ was HIF-1α dependent; we used lentiviral shRNA expression systems specifically targeting HIF-1α gene in H-Ras^V12^ expressing HDFs (Figures 3A, B and S1). Here we show that the suppression of HIF-1α activity restored the ability of H-Ras^V12^ to induce certain hallmarks of senescence namely p53 and p21^CIP1^ in HDF cells (Figure 3C). Interestingly, expression of p16^INK4a^ was not restored after HIF-1α knock-down (Figure 3C). Moreover, upon knock down of HIF-1α we also detected a significant decrease in expression of MIF under hypoxic conditions indicating hypoxic induction of MIF is HIF-1α dependent. Thus, there results show that induction of HIF-1α directly triggers the hypoxic down regulation of p53 and p21^CIP1^ but not p16^INK4a^. Moreover, our observation on HIF-1α dependent induction of MIF in hypoxia suggests the possibility that the decrease in expression of p16^INK4a^ in hypoxia was regulated through mechanisms alternative to MIF.

### Knockdown of HIF-1α induces apoptosis in H-Ras^V12^expressing HDFs in hypoxia

**Figure 4:**
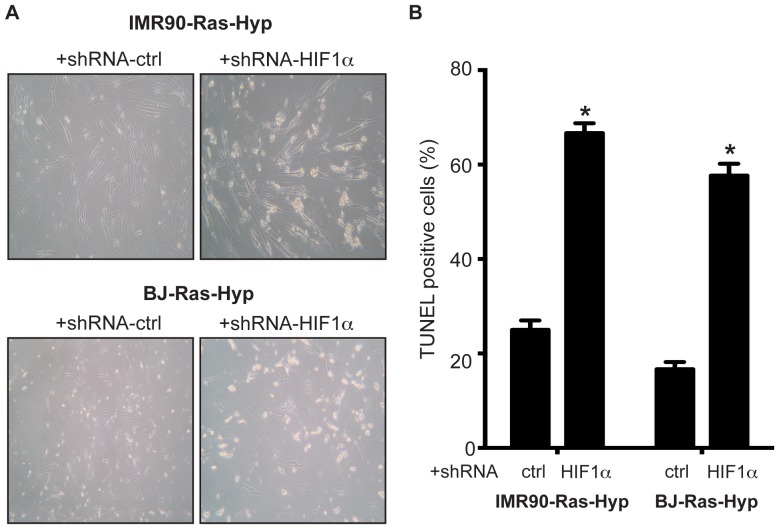
Knock down of HIF-1α in hypoxic conditions results in induction of apoptosis. shRNAHif-1α and H-Ras^V12^ as well as shRNANC (Negative control) and H-Ras^V12^ expressing IMR-90 and BJ cells were exposed to hypoxia. Three days post exposure to hypoxia (1%O_2_) cells were **A**. photographed with bright field microscopy; **B**. collected and fixed for TUNEL staining. *Columns*, mean of three independent experiments done in triplicate show percent increase in the number of TUNEL-positive cells; *bars*, SD. apoptosis is shown. *Columns*,means ± SD of three independent experiments done in triplicate. For statistical analysis the Student's *t*-test was performed comparing specific apoptosis of Ras + shNC *vs*. Ras+ shHIF-1α expressing cells in hypoxia (**p<0.01).

One of the hallmarks of OIS is the critical involvement of p53 and p16^INK4a^ -RB pathways. Since we found that knock down of HIF-1α can restore p53 and p21^CIP1^ expressions, we aimed to investigate whether senescence can be reinstated under these conditions. Surprisingly, after knock down of HIF-1α in H-Ras^V12^-expressing HDFs cultured under hypoxic conditions, substantial amount of cell death was detected within 3 days (Figure 4A). This finding was confirmed by TUNEL staining as apoptosis (Figures 4B and S2). Taken together our data indicate knockdown of HIF-1α leads to induction of apoptosis, but not restoration of senescence in H-Ras^V12^ expressing HDFs under hypoxia.

### Culturing under hypoxic conditions impairs H-Ras^V12^ -induced DNA damage response (DDR)

Recent research has shown causal connection of DNA damage response (DDR) and oncogene-induced senescence [8], [9]. These findings have underscored the importance of intact DDR machinery on a single cell level as a response to oncogene activation in senescence induction. Our hypothesis was that hypoxic conditions, consequently leading to the induction of HIF-1α activity, might have an impact on oncogene-induced DDR in HDF cells, and through that impact the initial steps of senescence induction.

**Figure 5:**
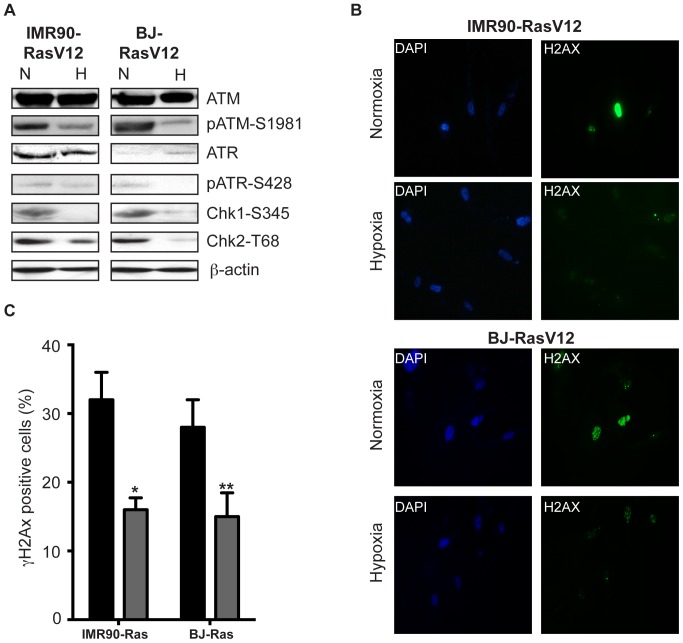
H-Ras^V12^ overexpression in hypoxic moiety down regulates DNA damage response (DDR). DNA damage signaling pathway in H-Ras^V12^-induced senescence in BJ and IMR-90 cells after 10 days exposure to **N** (normoxia, 20% O_2_) or **H** (hypoxia, 1% O_2_) **A**. Immunoblot analysis for total ATM and ATR, as well as ATM, ATR, Chk1 and Chk2 phosphorylations on Ser1981, Ser428, Ser296, Thr68, respectively. β-actin was used as loading control; **B**. Immunofluorescence analysis for γH2AX foci; DAPI was used to counterstain nuclei **C**. Quantification of the number of γH2AX foci. Histogram indicates the number of cells containing 5–10 foci. Black bars normoxia (20% O_2_), grey bars hypoxia, (1% O_2_). The data represent the average and standard deviation of three independent counts of 100 cells each. For statistical analysis the Student's *t*-test was performed comparing of Ras expressing cells in normoxia (N) *vs*. in hypoxia (H), (* represents p<0,05, ** represents p<0,01).

For this purpose we have analysed levels and activity of DDR markers in H-Ras^V12^ expressing HDFs under either normoxic or hypoxic conditions. In hypoxia, we observed a significant decrease in p-ATR-S428 levels in BJ fibroblasts whereas this decrease was very modest in IMR-90 cells (Figure 5A). Levels of p-ATM-S198 were reduced in HDFs cultured under the hypoxic conditions (Figure 5A), accompanying the decrease in levels of both pChk1-S296 and pChk2-T68 (Figure 5A). In addition, we show here on a cellular level the decrease of DDR marks in hypoxia, as visualized by γH2AX (Figures 5 B–C and S2).

Taken together, we can conclude from the data shown in Figure 5 that upon the exposure of H-Ras^V12^ expressing HDFs to hypoxic environment, significant decrease in DNA damage response (DDR) occurs, as shown by multiple markers.

## Discussion

This study shows that hypoxic conditions provide the right environment for the suppression of H-Ras^V12^ induced senescence in human diploid fibroblast (HDFs) cells BJ and IMR-90. Moreover, the same conditions are promoting proliferation that was blocked under normal conditions upon H-Ras^V12^ expression. We show here that these mechanisms are executed in a HIF-1α dependent manner.

In our work we provide a number of findings supporting this conclusion. First we show that H-Ras^V12^ expressing HDFs grown under hypoxic conditions failed to senesce, but proliferated further during the period of 10 days. This finding was evidenced by a number of senescence hallmarks such as decreased SA-β-galactosidase activity, and decreased H3K9me3 marks, increased Ki67 positivity as well as increased BrdU incorporation. Second, when cultured in hypoxia, cells displayed a strong decrease in expression of senescence hallmarks such as p53, p21^CIP1^, p16^INK4a^ and as well as phosphorylated Rb, accompanied by induction of HIF-1α and MIF expression. Genetic knock down of HIF-1α showed that hypoxic down regulation of p53, p21^CIP1^ and MIF was HIF-1α dependent, whereas p16^INK4a^ was independent of HIF-1αactivity. Accordingly we found that restoration of p53 and p21^CIP1^ levels were not sufficient to reinstate senescence, but rather induced apoptosis under hypoxic conditions. This finding is, implicating p16^INK4a^ as an important, and perhaps the major factor of not only the induction, but also the maintenance and restoration of H-Ras^V12^ induced senescence.

In addition, we show that hypoxic conditions lead to decrease in marks of H-Ras^V12^-induced DNA damage response (DDR) in human diploid fibroblasts, as shown by decreased levels of phosphorylated versions of ATM, ATR, Chk1 and Chk2. We assume that in hypoxic environment most likely different set of molecules are involved in regulation of p16^INK4a^ axis of senescence-induction and/or maintenance; in this setting HIF-1α might be crucial for providing negative feedback by targeting p53-p21^CIP1^ axis in HDFs. It would be of a great importance for future work to investigate the interaction partners of p16^INK4a^ under hypoxic conditions.

Cellular senescence is an irreversible growth arrest state induced *via* signals triggered by telomere shortening (replicative senescence) or *via* different stimuli including activation of certain oncogenes (e.g. Ras, BRAF), inactivation of tumor suppressor gene (e.g. Pten), mitogenic stimulation, DNA damaging agents and oxidative stress [27]–[30]. Senescence, which is induced in primary cells *via* activation of mitogenic oncogenes such as Ras/BRAF (oncogene-induced senescence), acts as an initial barrier preventing normal cells transformation into a malignant cell [28], [29]. Regulation of senescence is mainly driven by p16^INK4a^-Rb and p14/p19^ARF^-p53 pathways or alternatively through different mechanisms including DNA damage signalling, involving activation of cell cycle checkpoint kinases ATM/ATR [2], [8]. Recent studies point out tissue hypoxia as another important factor involved in regulation of senescence though, most of the *in vitro* data studying senescence collected so far has been produced under hyperoxic conditions.

During the last years, number of studies has demonstrated that hypoxia can prevent replicative senescence [21], [31], and this is also valid for anticancer drug- or oncogene- induced senescence, in human or mouse cells, respectively [15]–[17]. Hypoxia induced prevention of replicative senescence is attributed to decreased DNA damage in mouse cells or reactive oxygen species (ROS) activated HIF-1α activity and its target human telomerase reverse transcriptase (hTERT) in human cells [15]–[17]. A recent study conducted with mouse embryonic fibroblasts (MEFs) showed that HIF-1α plays a critical role in delaying the onset of senescence *via* transcriptional activation of MIF and inhibition of p53-mediated pathways [15]. Likewise, exposure to hypoxic conditions reduce the levels and the extent of drug-induced senescence in cancer cells, in a HIF-1α dependent manner [17]. These studies underscore the importance of HIF-1α in regulation of replicative and drug- induced senescence under hypoxic conditions, which is normally found in large portions of tumor tissue found in all the mammals. We consider that one of the most important implications of senescence regulation by hypoxic environment is its impact on oncogene-induced senescence as it is crucial for the initial steps of tumor suppression.

Oncogene-induced senescence (OIS) is a failsafe programme acting as an important barrier in prevention of oncogenic transformation, thereby exerting the tumor suppressive role [28]. In primary fibroblasts, when OIS is activated through the overexpression of H-Ras, cells rapidly accumulate increased amounts of tumor suppressors p53 and pRb, and the downstream effectors such as p21^CIP1^ and p16^INK4a^ [5]. It has been shown previously in mouse embryonic fibroblasts (MEFs) that H-Ras-induced senescence is dependent on reactive oxygen species (ROS) production and it might be rescued under hypoxic conditions, through the decrease of ROS generation due to the limited oxygen levels [20]. However, other studies have shown contradictory data in primary mouse embryonic fibroblasts (MEFs), indicating the levels of intracellular ROS might increase under hypoxia and that the generation of ROS is necessary for hypoxic activation of HIF-1α, which in turn drives essentially extension of replicative lifespan [16]. Therefore, modulation of ROS by oxygen levels and/or the role of ROS on modulation of senescence during hypoxia remain highly controversial. Our data in HDFs indicates that H-Ras^V12^-induced senescence is blocked in low oxygen environment (hypoxia), which is in agreement with the previous publication of Lee and colleagues [20]. In addition, we show here that hypoxia induced inhibition of senescence is associated with HIF-1α dependent p53 and p21^CIP1^ down regulation and decreased DNA damage response. Recent studies described direct interactions between HIF-1α and p53 proteins, mostly through promoting p53 stabilization or HIF-1α degradation [32], [33]. Ultimately, p53 and HIF-1α targets have also been found to cross-regulate each other [34], [35]. It has been shown in replicative senescence that HIF-1α target MIF can directly bind and inhibit p53 and its target p21^CIP1^ [15]. Collectively, our data on HIF-1α dependent down regulation of p53 and p21^CIP1^ in HDFs in hypoxic environment is consistent with the above report.

p16^INK4a^ is an important regulator of Ras-induced senescence, primarily acting through the Rb axis [2]. The role of p16^INK4a^ in senescence induction is well documented [5], [36], [37] though data from these studies were produced in normoxic conditions, as well. We show here that p16^INK4a^ protein expression is down regulated in HDFs under hypoxia, independent of HIF-1α and its target MIF. A, previous report showed that the expression of p16^INK4a^ was down regulated under hypoxia/anoxia (0,1%O_2_), which was dependent on constitutive activation of PI3K/Akt and phosphorylation of GSK3β in cancer cells [38]. Consistent with these reports we propose here that other transcription factors normally activated in hypoxia may be also involved in hypoxia-dependent regulation of p16^INK4a^. In addition the severity of hypoxic condition or cell type may also affect the hypoxia dependent modulation of p16^INK4a^ expression. Our knowledge of p16^INK4a^ and its regulation under hypoxic environment is currently limited and further investigations are underway to elucidate the possible mechanisms.

According to recent studies, cells cultured under hypoxic conditions may acquire ability to prevent senescence through HIF-1α's central role and loss of HIF-1α in hypoxia or even in normoxia restores the cell's ability to reinstate senescence [17]. Interestingly, in HDFs knock down of HIF-1α did not reinstate H-Ras^V12^ induced senescence but instead induced cell death under hypoxic conditions. Previous reports indicate that regulation of cellular senescence is different between human and mouse cells, suggesting that the results obtained in a mouse model may not be necessarily valid for human cells [39]. One of the hallmarks of OIS is the critical involvement of p53-p21^CIP1^ and p16^INK4a^–pRb pathways. Indeed, inactivation of p53 or its upstream regulator, p14/p19^ARF^ is sufficient to bypass H-Ras^V12^-induced senescence in murine cells [5], whereas p16 ^INK4a^ seems more critical than p53 in human cells, as some cells depend exclusively on p16^INK4a^ for completing OIS. For example, normal human fibroblasts deficient for p16^INK4a^ are refractory to the senescence induction by H-Ras^V12^ [40]. Similarly, oncogenic H-Ras^V12^ did not cause senescence in freshly isolated human fibroblasts expressing low amounts of endogenous p16^INK4a^ [37]. Mechanisms of OIS do not seem to be fully identical between the cell types and diverse genetic contexts. This can be also exemplified by the signaling pathways transducing OIS in H-Ras^V12^ versus BRAF^E600^: H-Ras^V12^-induced senescence can be bypassed by functional inactivation of the p16^INK4a^–RB pathway, [5] whereas BRAF^E600^-triggered senescence cannot [29]. On the other hand oncogenic Ras may exert both proapoptotic and anti-apoptotic effects depending on the eminence of Ras effector pathway and the apoptotic machinery [41], [42]. In different studies, it has been reported that oncogenic Ras signaling *via* RAF pathway may generate apoptotic response mediated by p53 [42]-[44]. Thus, according to our data we suggest that the reinstatement of H-Ras^V12^ induced senescence in human diploid fibroblasts (HDFs) under hypoxic environment might depend on restored expression of p16^INK4a^. Further, we cannot rule out the possibility that increased expression of Ras and p53, but lack of HIF-1α, which (amongst other things) exerts anti-apoptotic effects in hypoxia, may favor the induction of apoptosis instead of senescence in HDFs.

Recent studies have shown the involvement of DNA damage signaling through ATM/ATR kinases as a crucial mediator of oncogene induced senescence [8], [9], [11], [12]. However, studies reporting preventive role for hypoxia on induction of senescence did not considerably elucidate regulation of DNA damage response (DDR) under hypoxic conditions or whether it is involved in hypoxia dependent suppression of senescence. In a recent report, hypoxia did not decrease levels of DDR and cell cycle arrest caused by etoposide in immortalized human fibroblasts [17]. On the other hand, extremely low levels of hypoxia (<0.1% O_2_) have been found to induce DDR, involving both ATR- and ATM-mediated signaling. Consequently hypoxia-induced DDR triggers p53-dependent apoptosis [45]. Our data suggest moderate down regulation of DDR under hypoxic conditions in H-Ras^V12^ expressing HDFs, to best of our knowledge this is the first comprehensive data elucidating the effects of hypoxia on OIS and DDR together. An effort is underway to fully understand the mechanism(s) of down regulation of DDR under hypoxic conditions.

In conclusion our data reveal that hypoxia can prevent H-Ras^V12^ -induced senescence in HDFs by down regulating hallmarks of senescence such as p53, p21^CIP1^ and p16^INK4a^, the latter seems to be indispensable for this response. In addition, at molecular level HIF-1α activity is most likely a requirement for promotion of this response due to its negative regulation on p53 and p21^CIP1^. These findings might indicate the mechanism by which hypoxic environment helps premalignant cells to evade impingement of cellular failsafe pathways.

## Supporting Information

Figure S1 **Raw RT-PCR data** (belonging to the histograms in Figure 2C and 3B).
(TIF)

Figure S2 **Raw TUNEL data** (belonging to histogram in Figure 4B), **raw H2AX foci data** (belonging to histogram in Figure 5C) **and raw BrdU data** (belonging to histogram in Figure 1D). Vector expressing cell values are normalized to control cell values and Ras expressing cell values are normalized to vector expressing cells.
(TIF)
