# Correction: The Role of Hypoxia Inducible Factor-1 Alpha in Bypassing Oncogene-Induced Senescence

**DOI:** 10.1371/journal.pone.0110981

**Published:** 2014-10-08

**Authors:** The PLOS ONE Staff

The original orientation of the western blot in Figure 3A was incorrect. The authors have provided a corrected version of Figure 3 here.

**Figure 3:**
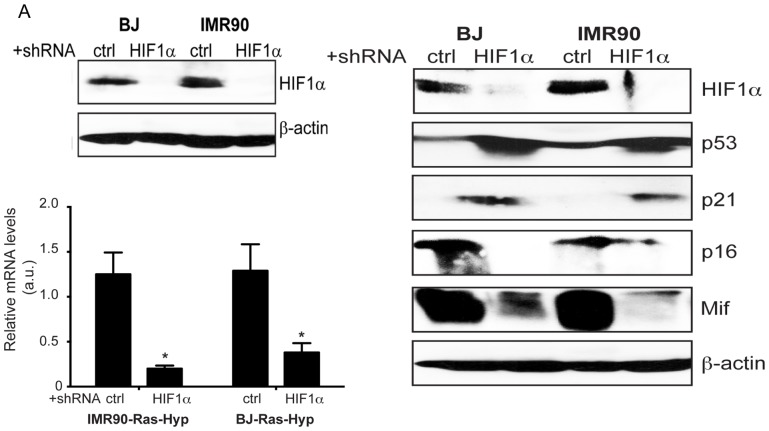
Hypoxia-driven inhibition of expression of hallmarks of senescence is Hif-1α dependent. shRNA_HIF-1α and shRNA_scr (a scrambled shRNA sequence encoding plasmid used as negative control) expressing IMR-90 and BJ cells were infected with H-RasV12 and selected for puromycin for 3 days. Three days post exposure to hypoxia cells were analyzed for A. the expression of HIF-1α by western-blotting, β-actin was used as loading control; B. for mRNA level by Quantitative RT-PCR; C. the expression of senescence regulators p53, p21CIP1, p16INK4a and MIF by western-blotting. β-actin was used as loading control. Statistically significant differences between mRNA levels of HIF-1α in Ras + shNC vs. Ras+ shHIF-1α expressing cells in hypoxia are indicated *, p<0.01. Shown are means ± SD of 3 independent experiments in triplets.

## References

[1] Kilic Eren M, Tabor V (2014) The Role of Hypoxia Inducible Factor-1 Alpha in Bypassing Oncogene-Induced Senescence. PLoS ONE 9(7): e101064 doi:10.1371/journal.pone.0101064 24984035 10.1371/journal.pone.0101064 PMC4077769

