# Supplementary figures and images for: The Role of Hypoxia Inducible Factor-1 Alpha in Bypassing Oncogene-Induced Senescence
Source: PLoS One. 2014 Jul 1;9(7):e101064. doi: 10.1371/journal.pone.0101064 (PMC4077769; doi:10.1371/journal.pone.0101064)

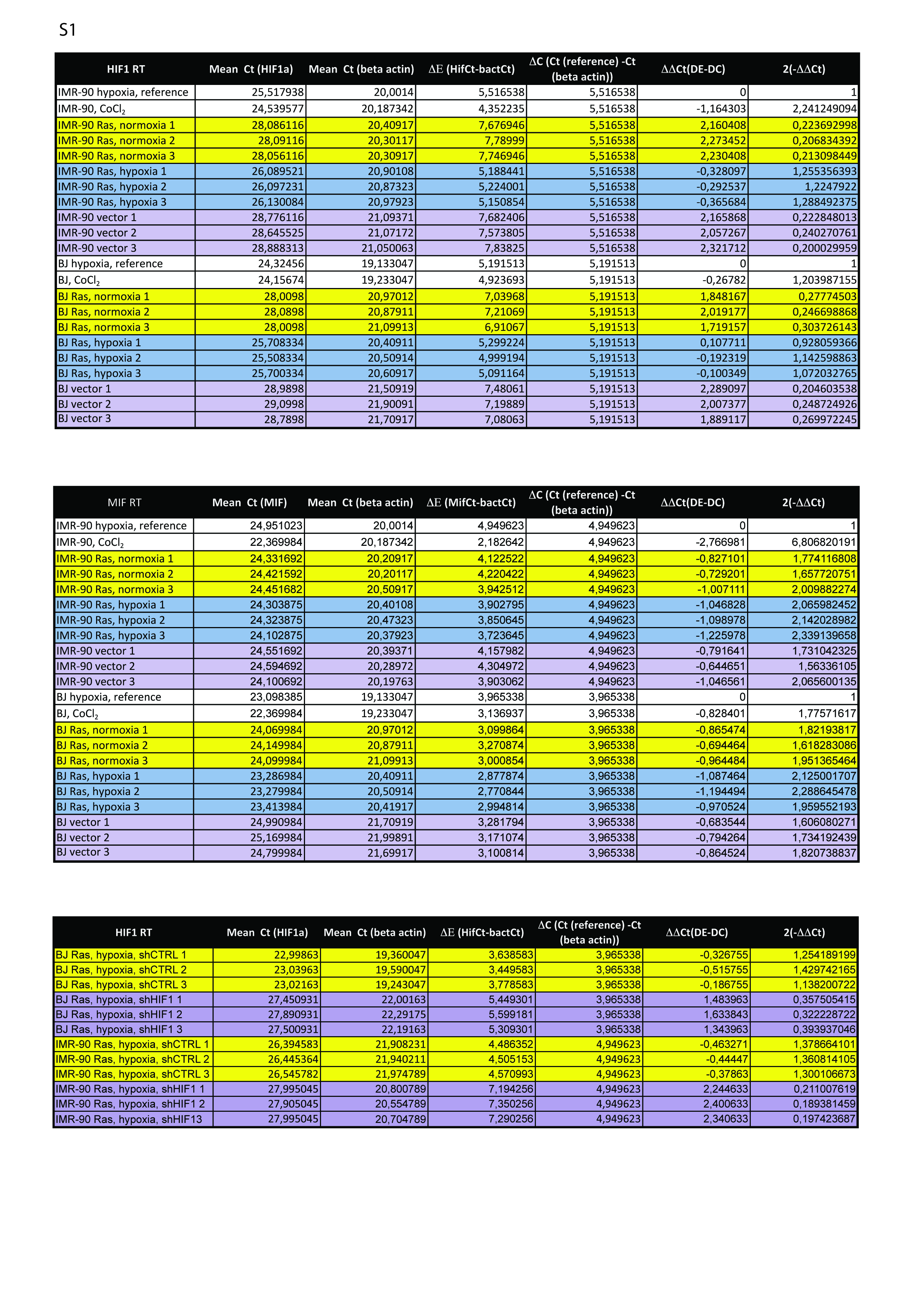

Supplement: Figure S1 — Raw RT-PCR data (belonging to the histograms in Figure 2C and 3B). (TIF) [file pone.0101064.s001.tif]

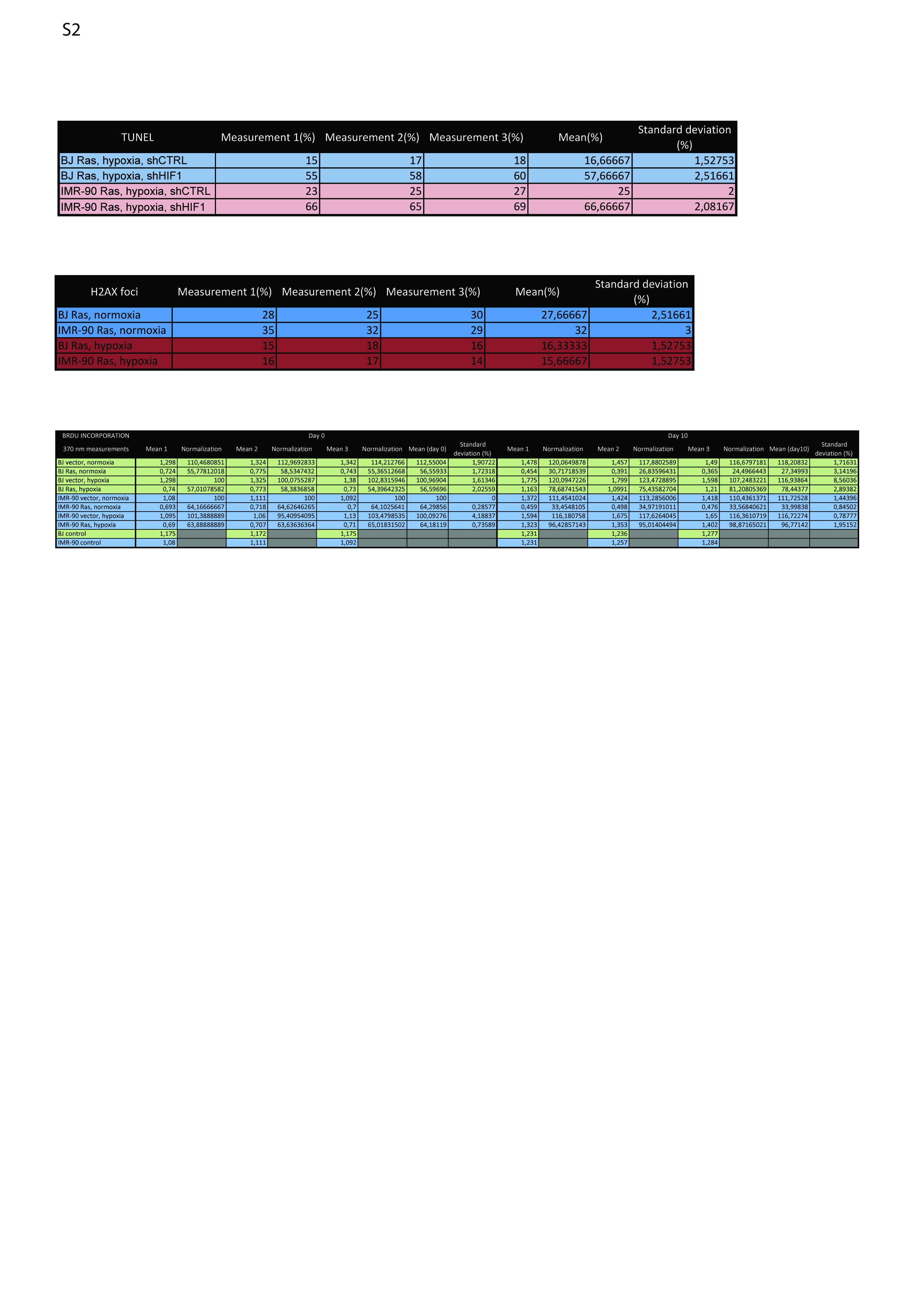

Supplement: Figure S2 — Raw TUNEL data (belonging to histogram in Figure 4B), raw H2AX foci data (belonging to histogram in Figure 5C) and raw BrdU data (belonging to histogram in Figure 1D). Vector expressing cell values are normalized to control cell values and Ras expressing cell values are normalized to vector expressing cells. (TIF) [file pone.0101064.s002.tif]
